# Vaccination against COVID-19 and Clinical correlates among a population of psychiatric outpatients

**DOI:** 10.1192/j.eurpsy.2023.1687

**Published:** 2023-07-19

**Authors:** M. jabeur, L. Gassab, B. Ben Mohamed, A. Ben Hawala, A. Mhalla, F. Zaafrane, L. Gaha

**Affiliations:** Psychiatry, University Hospital of Monastir, Monastir, Tunisia

## Abstract

**Introduction:**

Patients suffering from psychiatric disorders represent a population that is particularly at risk of COVID-19-related morbidity and mortality. Vaccination was the most effective strategy to prevent the severe forms of the disease.

**Objectives:**

We aimed in our study to determine the rate of COVID-19 vaccination and to identify its correlated factors in psychiatric outpatients.

**Methods:**

This is a descriptive and analytical cross-sectional study conducted on 178 outpatients at the department of psychiatry (Monastir, Tunisia) over a period of one month (from March 2022 to April 2022). Data was collected via a questionnaire focused on two main attributes: (1) sociodemographic and clinical characteristics; (2) questions about the flu vaccination history and its modalities.

**Results:**

The mean age of our patients was 44.9±13.7 years. The majority of them (81.5%) had a chronic evolution of their psychiatric disorder (> 2 years). Psychosis was the most represented disorder with 57.3% compared to mood disorders and anxiety disorders. Among our population, 73% of the patients received vaccination against COVID-19. The majority got 2 doses (60%), were vaccinated on their own initiative (68%) and by making an appointment (71.4%). Patients with depressive disorders accessed to vaccination program in 100% of cases. The group of psychotic patients had a vaccination rate of 66%. Vaccination was significantly associated with gender (p=0.001), age (p=0.04), marital status (p<10-3), number of children (p=0.002), housing situation (0.018), diagnosis (p<10-3) and treatment (p=0.01)

**Conclusions:**

Patients with psychiatric disorders experience a distinct burden of the COVID-19 disease. They should therefore be prioritised in vaccine allocation strategies, especially among patients with psychotic disorders.

**Disclosure of Interest:**

None Declared

